# Polyangiitis with Granulomatosis as a Paraneoplastic Syndrome of B-Cell Lymphoma of the Lacrimal Gland

**DOI:** 10.1155/2014/713048

**Published:** 2014-12-18

**Authors:** Beatriz Wills Sanín, Yenny R. Cárdenas Bolivar, Jose J. Carvajal, Guillermo E. Quintero, Rafael Andrade

**Affiliations:** ^1^Departamento de Critical Care, Hospital Universitario Fundación Santa Fe de Bogotá, Calle 119 No. 7-75, Bogotá, Colombia; ^2^Departamento de Hematology, Hospital Universitario Fundación Santa Fe de Bogotá, Bogotá, Colombia; ^3^Departament of Pathology, Hospital Universitario Fundación Santa Fe de Bogotá, Universidad de los Andes, Bogotá, Colombia

## Abstract

*Introduction*. The clinical course of an autoimmune paraneoplastic syndrome parallels the natural history of the primary malignancy. In most cases, such paraneoplastic are syndromes hardly distinguishable from idiopathic autoimmune diseases. A case of polyangiitis with granulomatosis as a paraneoplastic syndrome in a patient with B-cell Lymphoma of the lacrimal gland has not yet been reported. *Case Presentation*. We present the case of a male patient with a B-cell Lymphoma of the lacrimal gland, who debuted with symptoms similar to rheumatoid arthritis and acute renal failure, secondary to polyangiitis with granulomatosis. The current pathophysiological hypotheses explaining the relationship between a lymphoproliferative disease and an autoimmune paraneoplastic disorder are discussed. *Conclusion*. Tumor-associated segmental necrotizing glomerulopathy is a very rare manifestation of glomerular diseases. Some atypical clinical features should increase the suspicion of an underlying tumor, in which case it is essential to treat the primary neoplasia, in order to control the autoimmune manifestations.

## 1. Introduction

Paraneoplastic syndromes occur in approximately 10% of all malignant tumors. Their presence can complicate the diagnosis and treatment of the underlying malignancy. Some rheumatologic disorders are associated with, or may be preceded by, the clinical manifestations of various solid and hematological tumors [1]. Identifying the expression of various rheumatic diseases is essential for the early diagnosis and effective treatment of cancer. A high degree of suspicion on the relationship between these pathologies allows the clinician to control the inherent life-threatening complications and increased risk of mortality. Many cases have been described, but a pathophysiological explanation remains to be elucidated [2].

## 2. Case Report

A 54-year-old male developed progressive right conjunctival injection, diplopia, and edema in the upper eyelid, of two-month duration. Additionally, he complained of fatigue, dry mouth, orthostatism and gingivorragia after brushing his teeth, persistent dry cough, ageusia, decreased appetite, and unintentional weight loss of 6 kg in the past two months. The patient's relevant past medical history included a diagnosis of rheumatoid arthritis in his father and sister, as well as a 15-pack-year smoking history.

**Figure 1:**
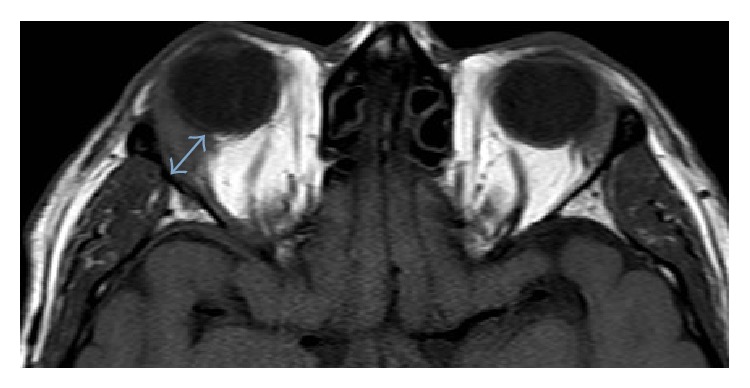
Contrast enhanced axial T2 weighted image showing enlarged right lacrimal gland (blue arrow) with soft tissue compromise suggestive of chronic inflammation.

**Figure 2:**
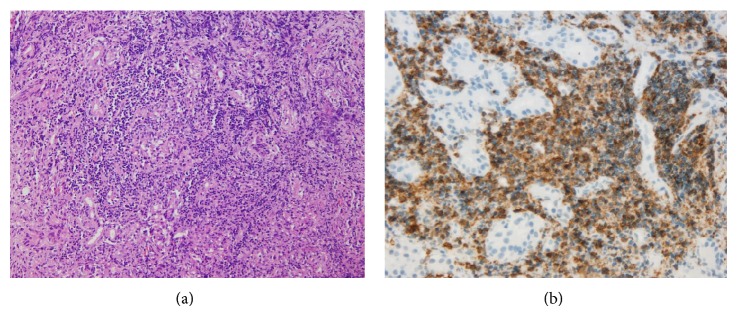
Orbit (a) histological appearance with diffuse lymphoid infiltrate H&E stain 10x and (b) immunohistochemistry study showing uniform B-cell population CD20, 40x.

**Figure 3:**
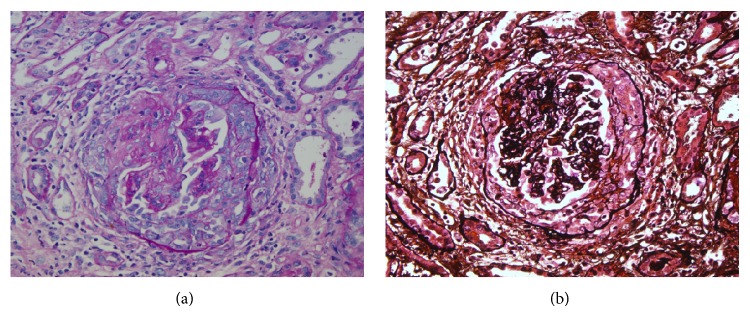
Glomerulus with extracapillary proliferation. Left side with PAS stain, 20x, and to the right with methenamine-silver stain, 20x.

An orbital MRI was ordered to study the ophthalmological symptoms (Figure 1), which showed changes suggestive of a chronic, granulomatous inflammation in the right lacrimal gland. Histopathological studies (Figures 2 and 3) revealed a predominantly B, lymphoid, and small-cell infiltrate. Phenotypically, the cells were CD20-, CD43-, and CD23-positive with excess of lambda stain on the B-cells; without expression of CD10 and CYCLIN D, the proliferative activity, as measured by Ki67, was estimated to be 10%. This morphological and phenotypical pattern, with a predominance of lambda expression, favored a marginal zone B-cell lymphoma of the lacrimal gland. Extension studies, including a thoracoabdominal CT and bone marrow biopsy, were negative for tumor involvement. A course of R-CVP (rituximab, cyclophosphamide, vincristine, and prednisone) chemotherapy was initiated.

Before starting chemotherapy, the patient presented Raynaud's phenomenon and predominantly asymmetric and migrating polyarthralgia in small- and medium-sized joints, for which he was evaluated by rheumatology. Laboratory tests showed a positive c-ANCA (cytoplasmic antineutrophil cytoplasmic antibodies) titer of 1 : 80 (less than 1 : 20 is negative) and positive antiproteinase antibodies at 5.8 U (normal values < 0.4 U). Due to these findings, the arthralgias were considered of paraneoplastic origin and were initially managed with a short cycle of anti-inflammatory drugs. Subsequently, the patient presented to the emergency room complaining of 3 days of hematochezia. Upon admission, the patient was in regular general condition, with tachycardia, hypotension, and an adequate ventilatory pattern. Physical examination revealed pain upon palpation of elbows, hand, and ankle joints. The remainder of the physical exam was within normal limits.

The patient underwent emergency dialysis due to severe metabolic acidosis, a marked elevation of blood nitrogen, and hypercalcemia (with impact on the electrocardiogram, which showed peaked T waves and shortening of the QT interval) and was transferred to the intensive care unit. Working diagnosis included renal failure secondary to vasculitis or chronic use of nonsteroidal anti-inflammatory drugs. A renal biopsy showed segmental necrotizing glomerulonephritis (Figure 2) with 60% and 21% cellular and fibrous extracapillary proliferation, respectively, compatible with type 3 extracapillary proliferative glomerulonephritis (type 3, pauci-immune). Severe acute interstitial nephritis was also observed, with eosinophils, neutrophils, and severe atherosclerosis. These studies, along with the clinical history, suggest a rapidly progressive glomerulonephritis. In consensus with oncology, hematology, and nephrology, cyclophosphamide was begun as treatment for the vasculitis and as part of a chemotherapy cycle to treat his lacrimal gland lymphoma, whereupon the systemic symptoms resolved.

## 3. Discussion

Lymphoproliferative and autoimmune disorders share signs and symptoms that may confound the clinical presentation of these two entities, which may, in fact, coexist. Indeed, it is not uncommon to find patients with chronic lymphocytic leukemia and autoimmune hemolytic anemia or lymphoma and Sjogren's syndrome. The pathogenesis of paraneoplastic rheumatic diseases is complex and is not yet fully understood [3].

It has been suggested that patients with autoimmune diseases can develop lymphoproliferative disorders secondary to antigenic stimulation, as observed in inflammatory diseases such as celiac disease and mucosa associated lymphoid tissue (MALT) lymphomas. Their manifestations may also correspond to an underlying, often hidden, cancer caused by a wide variety of tumor effects that are not related to metastasis or to the mechanical impact of the tumor mass, but rather as a result of substances released by tumor cells, such as hormones, peptides, antibodies, and other immunological reactions related to the host. Thus, rheumatic syndromes may be important clues to suspect a hidden neoplasia [4].

In general, rheumatological symptoms precede the diagnosis of cancer and therefore serve as a prognostic factor for malignancy. It is important to recognize which clinical signs suggest an occult neoplasm, such as acute onset of symptoms, age outside the usual ranges, poor response to corticosteroids, atypical distribution of articular involvement, and abnormal lab results (persistent anemia, thrombocytopenia, hypergammaglobulinemia, and hidden blood in stool). Treatment of autoimmune symptoms may mask the manifestations of lymphoma and delay the diagnosis or even induce resistance to chemotherapy due to chronic exposure to suboptimal doses of chemoimmunotherapy [5].

Patients who have autoimmune diseases and cancer can be classified into three groups: in the first, the autoimmune disease may be triggered by a tumor or metastasis. This occurs in arthritis with synovial infiltration by leukemic cells. The second class of patients refers to those who have an autoimmune disease and who subsequently have cancer, with an interval of up to 20 years, for example, Sjogren's syndrome and lymphoma. The third group includes patients with clinical manifestations of an autoimmune disease as an expression of a hidden cancer, that is, a paraneoplastic syndrome [6].

These relationships have stimulated research in rheumatology and oncology in order to understand the pathophysiological relationship between cancer and rheumatic manifestations. Three hypotheses have been described as follows: (i) both malignancies and paraneoplastic syndrome have independent effects and a common etiology, such as a viral infection or physical stimuli such as ultraviolet radiation; (ii) paraneoplastic autoimmune manifestations are a direct effect of toxicity triggered by tumor cells that stimulate tissue inflammation and, thus, classic rheumatologic symptoms; (iii) paraneoplastic autoimmune entities are mediated by hypersensitivity reactions due to the expression of tumor antigens shared by target cells of autoimmune diseases, including proteins associated with the tumor cell's nucleic acids. This last hypothesis is based on the demonstration of autoantibodies against nuclear proteins and double-stranded DNA in the serum of patients with autoimmune paraneoplastic syndromes [7].

Different clinical presentations have been shown as part of a paraneoplastic rheumatic syndrome, such as connective tissue diseases, rheumatoid arthritis-like syndrome, polymyalgia rheumatica, and vasculitis. These patients generally present with asymmetrical polyarthritis—such as the case presented here, which can be mistaken for rheumatoid arthritis or seronegative spondyloarthropathies. Clinical manifestations include acute onset and predominance in legs without compromising the small joints of the hands, nonspecific synovitis with normal X-rays, and the absence of rheumatoid factor. In these circumstances, disproportionate pain, marked weight loss, hepatosplenomegaly, lymph node enlargement, a poor response to steroids and disease-modifying antirheumatic drugs, and the presence of unexplained anemia should prompt a more extensive investigation [8].

Several studies have shown an increase in the appearance of malignant tumors in patients with autoimmune diseases. Solid epidemiological evidence attests that dermatomyositis and polymyostis may present as paraneoplastic syndromes. According to the literature, the incidence of association of malignant diseases to myositis varies between 7% and 66% [5]. It has also been reported that patients with polyangiitis with granulomatosis have a four- to eleven-fold increase in the risk of lymphoma. In most cases, this increase in cancer rate has been related to the use of immunosuppressive therapy. It may take several years for rheumatic symptoms or for a tumor to be diagnosed; however, rapid-onset rheumatoid arthritis has been reported as one of the first manifestations of malignancy, as occurs in the case we are presenting [9].

The clinical course of rheumatic manifestations usually parallels that of cancer. Symptoms often do not respond to antirheumatic therapy, while radical treatment of the primary malignancy usually results in regression of paraneoplastic rheumatic syndrome, as was seen in our patient. Malignant tumors that have been associated with these syndromes include those in lungs, colon, breast, ovary, stomach, and oropharyngeal and hematopoietic system. Several studies were able to demonstrate that a rheumatic syndrome may precede the development of cancer on average by eight to twelve months [10].

Some symptoms similar to scleroderma and Raynaud's phenomenon may also occur within the spectrum of a paraneoplastic syndrome in lymphoma, multiple myeloma, and liver, kidney, or ovarian carcinoma. Warning signs for malignancy include Raynaud's phenomenon as an isolated symptom with a normal capillaroscopy, acute onset, age above 50 years, and asymmetric finger compromise, with rapid progression to necrosis or poor response to vasodilator therapy or sympathectomy. Both rheumatologic symptoms and secondary complications to vasospasm resolve once the patient is treated surgically or with chemotherapy [10].

Vasculitis is another common presentation of a paraneoplastic syndrome. Patients with polyangiitis and granulomatosis frequently have involvement of the peripheral nervous system, which arises in 15% to 40% of cases, usually in the form of multiple mononeuritis or mixed polyneuropathy. Most patients (83%) develop peripheral neuropathy in the first 2 years of disease, and this may be the first sign of polyangiitis. Skin lesions, such as digital ischemia with necrosis, can also be found. The incidence of malignancies in patients with vasculitis is estimated at 8%, and the cutaneous leukocytoclastic variant is most frequent. The types of cancer with a greater association to vasculitis are lymphoproliferative disorders and myelodysplastic syndromes. Vasculitis associated with c-ANCAS, such as polyangiitis with granulomatosis, appears with greater frequency in men older than 40. The interval between the onset of vasculitis and the diagnosis of the tumor, or vice versa, is on average between one and three months [1].

The clinical presentation of paraneoplastic vasculitis, and vasculitis in patients without malignancy, is similar. However, the age at diagnosis, the absence of a clear correlation with prior infection or systemic autoimmune diseases, a chronic-relapsing course, and a lack of response to conventional treatment are findings that are more suggestive of an occult neoplasm. Immune complexes and complement consumption are typically absent in autoimmune paraneoplastic syndromes [11].

Inflammatory phenomena, such as the secretion of proinflammatory cytokines, including tumor necrosis factor alpha, and the excessive activation of neutrophils and monocytes triggered by vasculitis can induce the appearance of vasculitis in the form of polyangiitis with granulomatosis. In addition, autoantibodies such as c-ANCAS can trigger a cross-reaction and stimulate the proliferation of lymphoid tissue, thereby increasing the risk of developing malignancies. Medical literature suggests that the risk of developing some type of neoplasia is higher in patients with polyangiitis with granulomatosis, with a 4 to 10 times greater risk of developing lymphoma. This association can be explained by the immunosuppressive treatment and the presence of ANCA [12].

The patient described had a family history of rheumatologic disease. Early rheumatologic symptoms (Raynaud's phenomenon and asymmetric and acute onset arthralgia) presented shortly after the diagnosis of lacrimal gland B-cell lymphoma. Despite the fact that symptomatic treatment was begun, it is likely that the same autoimmune phenomenon also led to the emergence of polyangiitis with granulomatosis. The genes associated with a susceptibility of developing polyangiitis with granulomatosis are encoded in a set of immunologically relevant molecules. Although we did not perform a genetic study on the patient we reported, there are different alleles, such as HLA-DPB1 and the retinoid receptor X (RXRB), two neighboring genes in the dense region of the Class II MHC, that confer an increased risk for developing polyangiitis with granulomatosis as a paraneoplastic syndrome [13].

Paraneoplastic glomerulonephritis is rare complication secondary to malignancies, characterized by glomerular lesions that are not directly related to tumor burden, invasion, or metastasis but is rather induced by products of the tumor cells. The relation between membranous nephropathy and solid epithelial tumors, and minimal change disease related to Hodgkin's disease, was described over 60 years ago. With advances in research, it has been possible to identify other entities, such as focal segmental glomerulosclerosis (FSGS), membranoproliferative glomerulonephritis, IgA nephropathy, the latter described in patients with tumors of the respiratory tract, oral mucosa, and nasopharynx, and rapidly progressive glomerulonephritis (RPGN), as paraneoplastic manifestations [14].

Solid tumors, mostly lung, gastric, kidney, and prostate carcinomas, may trigger paraneoplastic membranous nephropathy. Some clinical features described in these cases are male, age > 50 years, and less than 12 months between the nephropathy and cancer. The prevalence of cancer in a cohort of 240 patients with membranous nephropathy was 10%. The authors also identified that a smoking history of more than 20 pack-years helped to discriminate paraneoplastic membranous glomerulonephritis from the idiopathic type. It is common to observe a complete remission of the nephrotic syndromes in patients whose tumor is in remission [15].

In addition to finding subepithelial deposits of immune complexes, the pathology of paraneoplastic membranous nephropathy also includes an increase in the number of inflammatory cells, in comparison with idiopathic membranous nephropathy. Additionally, the IgG1 and IgG2 subtypes, associated with Th1 T-helper cells, are more frequent in the kidneys of patients with paraneoplastic membranous nephropathy than in those with idiopathic membranous nephropathy. On the other hand, the IgG4 isotype related to Th2 cells stimulated by IL-13 and IL-40 is strongly associated with proliferative and crescent forms, as well as paraneoplastic membranous nephropathy. This particular immune pattern that favors both Th1 and Th2 cytokines seems to be triggered by tumor antigens that increase the number of inflammatory cells, a situation that is characteristic of paraneoplastic glomerulonephritis [14].

Rapidly progressive glomerulonephritis, as diagnosed in our case report, can also be associated with solid tumors and lymphoma, and as previously mentioned, patients with antineutrophil cytoplasmic antibodies (ANCA) are at an increased risk of developing cancer, as compared to the general population. The literature recommended tumor ablation, as well as immediately beginning treatment with steroids and cyclophosphamide, in patients with paraneoplastic rapidly progressive glomerulonephritis in order to prevent irreversible renal damage. Henoch-Schonlein purpura is another form of systemic vasculitis associated with IgA nephropathy that can be related to solid tumors, especially lung cancer [12].

The relationship between paraneoplastic glomerulonephritis and chronic lymphoid neoplasms is widely known. Minimal change GMN is the entity that is most often associated with Hodgkin's lymphoma, occurring in 1% of patients. It is followed by the focal segmental GMN, and membranous nephropathy is commonly associated with chronic lymphocytic leukemia, non-Hodgkin's lymphoma, and hairy cell leukemia [14].

It has been reported that up to 71% of patients with Hodgkin's lymphoma and minimal change GMNs report systemic symptoms (fever, weight loss, and nocturnal diaphoresis), 90% of whom have high levels of Th2 cytokines and acute phase reactants. It is therefore justified to rule out Hodgkin's lymphoma in patients with minimal change GMN and the aforementioned features [11].

The prevalence of paraneoplastic glomerular diseases in patients with thymoma is approximately 2%. The most common paraneoplastic GMN, as in other hematological malignancies, is also minimal change GMNs. Similarly, thymoma polarizes the immune response towards a Th2 profile, favoring the development of glomerular lesions associated with thymoma and hematological malignancies. In addition, this trend towards proliferation of Th2 lymphocytes would allow for the development of biomarkers that separate paraneoplastic glomerulonephritis from secondary or idiopathic glomerulonephritis [13].

The most difficult issue in paraneoplastic glomerulonephritis is the clinical recognition of this syndrome. The results of a delay in diagnosis can be serious, since patients may be subjected to potentially harmful treatments. In patients who present with glomerulonephritis and preexisting malignancy, it is important to rule out glomerular injury induced by cancer treatment, such as thrombotic microangiopathy induced by mitomycin-C.

Tumor markers, including carcinoembryonic antigen (CEA), prostate-specific antigen (PSA), and melanoma antigens, have been associated with paraneoplastic membranous nephropathy; however, it has not been established whether or not these tumors directly trigger glomerular damage. It is still necessary to confirm whether the increase in the number of inflammatory cells and IgG1 and IgG2 staining are diagnostic of paraneoplastic membranous nephropathy and thus justify an exhaustive search for malignancies [14].

A multidisciplinary approach is necessary to identify and treat both the malignancy and the paraneoplastic glomerulonephritis.

## 4. Conclusion

It is important to recognize the association between lymphoproliferative disorders and autoimmune diseases, to ensure a proper clinical evaluation and timely treatment. This case highlights the importance of having a high level of suspicion of granulomatous vasculitis in the presence of lymphoma, since different rheumatic symptoms may be the initial manifestation of an occult cancer. We recommend performing renal biopsy in patients with glomerular proteinuria or nephrotic syndrome and cancer, according to the patient's life expectancy, to determine if it is in fact a paraneoplastic syndrome. It is still necessary to have a greater understanding of the clinical and pathophysiological features of the autoimmune paraneoplastic syndromes, which will allow the differentiation from a case of autoimmunity without an associated neoplasia, in order to guide investigations made in search of a primary neoplasia.

## Abbreviations

c-ANCA: Cytoplasmic antineutrophil cytoplasmic antibodies
CEA: Carcinoembryonic antigen
GMN: Glomerulonephritis
MALT: Mucosa associated lymphoid tissue
PSA: Prostate-specific antigen
R-CVP: Rituximab, cyclophosphamide, vincristine, and prednisone
RPGN: Rapidly progressive glomerulonephritis
VEGF: Vascular endothelial growth factor.

## References

[1] Racanelli V., Prete M., Minoia C., Favoino E., Perosa F. (2008). Rheumatic disorders as paraneoplastic syndromes. *Autoimmunity Reviews*.

[2] Chakravarty E., Genovese M. C. (2003). Rheumatic syndromes associated with malignancy. *Current Opinion in Rheumatology*.

[3] Aruch D. B., Mims M. P. (2013). Paraneoplastic nephrotic syndrome and inflammatory arthritis at diagnosis in Hodgkin lymphoma. *Clinical Lymphoma Myeloma and Leukemia*.

[4] Baimpa E., Dahabreh I. J., Voulgarelis M., Moutsopoulos H. M. (2009). Hematologic manifestations and predictors of lymphoma development in primary sjögren syndrome: clinical and pathophysiologic aspects. *Medicine*.

[5] András C., Csiki Z., Ponyi A., Illés A., Dankó K. (2006). Paraneoplastic rheumatic syndromes. *Rheumatology International*.

[6] Szekanecz Z., Szekanecz É., Bakó G., Shoenfeld Y. (2010). Malignancies in autoimmune rheumatic diseases—a mini-review. *Gerontology*.

[7] Fam A. G. (2000). Paraneoplastic rheumatic syndromes. *Best Practice & Research Clinical Rheumatology*.

[8] Baijens L. W. J., Manni J. J. (2006). Paraneoplastic syndromes in patients with primary malignancies of the head and neck. Four cases and a review of the literature. *European Archives of Oto-Rhino-Laryngology*.

[9] Solans-Laqué R., Bosch-Gil J. A., Pérez-Bocanegra C., Selva-O'Callaghan A., Simeón-Aznar C. P., Vilardell-Tarres M. (2008). Paraneoplastic vasculitis in patients with solid tumors: report of 15 cases. *Journal of Rheumatology*.

[10] Azar L., Khasnis A. (2013). Paraneoplastic rheumatologic syndromes. *Current Opinion in Rheumatology*.

[11] Bacchetta J., Juillard L., Cochat P., Droz J.-P. (2009). Paraneoplastic glomerular diseases and malignancies. *Critical Reviews in Oncology/Hematology*.

[12] Pankhurst T., Savage C. O. S., Gordon C., Harper L. (2004). Malignancy is increased in ANCA-associated vasculitis. *Rheumatology*.

[13] Lien Y.-H. H., Lai L.-W. (2011). Pathogenesis, diagnosis and management of paraneoplastic glomerulonephritis. *Nature Reviews Nephrology*.

[14] Jhaveri K. D., Shah H. H., Patel C., Kadiyala A., Stokes M. B., Radhakrishnan J. (2014). Glomerular diseases associated with cancer, chemotherapy, and hematopoietic stem cell transplantation. *Advances in Chronic Kidney Disease*.

[15] Lefaucheur C., Stengel B., Nochy D. (2006). Membranous nephropathy and cancer: epidemiologic evidence and determinants of high-risk cancer association. *Kidney International*.

